# Associations of serum sLOX-1 levels with disease severity and 3-month function prognosis after spontaneous intracerebral hemorrhage: a prospective cohort study

**DOI:** 10.3389/fneur.2025.1521774

**Published:** 2025-03-12

**Authors:** Xiufeng Ye, Heng He, Huan Song, Jing Huang, Zhixing Zhang, Yan Zhou

**Affiliations:** ^1^Department of Neurosurgery, Longquan People’s Hospital, Lishui, China; ^2^Department of Neurosurgery, Lishui City People’s Hospital, Lishui Hospital of Wenzhou Medical University, Lishui, China; ^3^Department of Neurosurgery, Jinyun County Hospital of Traditional Chinese Medicine, Yuncheng, China

**Keywords:** spontaneous intracerebral hemorrhage, biomarker, prognosis, soluble lectin-like oxidized low-density lipoprotein receptor-1, severity

## Abstract

**Background:**

Soluble lectin-like oxidized low-density lipoprotein receptor-1 (sLOX-1) may be involved in the inflammatory response and aggravate secondary brain injury after spontaneous intracerebral hemorrhage (sICH). The aim of this study was to reveal the association of serum sLOX-1 levels with disease severity and the predictive power of 90-day neurological outcomes after sICH.

**Method:**

This prospective cohort study included 118 sICH patients and 118 healthy controls, whose serum sLOX-1 levels were quantified. Glasgow Coma Scale (GCS) scores and hematoma volumes were used to assess disease severity. Glasgow Outcome Scale (GOS) scores were used to assess 3-month function prognosis after stroke. The relation of serum sLOX-1 levels to disease severity and prognosis (GOS scores 1–3) was discerned Receiver operating characteristic curve was built to evaluate the prognostic predictive capability.

**Result:**

Serum sLOX-1 levels were significantly increased in patients compared to healthy controls, and were independently correlated with GCS scores (*ρ* = −0.577, *p* < 0.001; *t* = −6.732, *p* < 0.001) and hematoma volumes (*ρ* = 0.540, *p* < 0.001; *t* = 7.136, *p* < 0.001). Patients with poor prognosis have higher serum sLOX-1 levels than in those with good prognosis (*p* < 0.001). Serum sLOX-1 levels >1539.75 pg/mL distinguished the risk of poor prognosis at 3 months after stroke, with a sensitivity of 83.72% and a specificity of 72.00% (area under curve, 0.813; 95% confidence interval (CI), 0.731–0.879, *p* < 0.001). Serum sLOX-1 levels were independently associated with poor 3-month prognosis with odds ratio of 1.002 (95% CI, 1.000–1.004).

**Conclusion:**

Serum sLOX-1 levels are obviously increased after stroke and are significantly associated with disease severity and poor prognosis. Hence, sLOX-1 may serve as a useful potential prognostic biomarker for sICH.

## Introduction

Spontaneous intracerebral hemorrhage (sICH) is a severe but less frequent form of stroke resulting from the spontaneous rupture of blood vessels in the brain, particularly affecting the elderly population. This condition is marked by rapid neurological deterioration and poor long-term prognosis (1). In clinical practice, disease severity and prognosis in sICH are primarily assessed using the Glasgow Coma Scale (GCS) and hematoma volume measurements (2). However, these methods have limitations in capturing the underlying pathophysiological mechanisms, driving an increasing interest in the use of biomarkers for more precise prognostic evaluation.

Lectin-like oxidized low-density lipoprotein receptor-1 (LOX-1) belongs to the C-type hemagglutinin family and is primarily expressed in endothelial cells (3, 4). Functioning as a membrane-bound protein, LOX-1 plays a pivotal role in endothelial cell endocytosis and the degradation of Ox-LDL. This, in turn, triggers various inflammatory responses and subsequently leads to endothelial cell dysfunction, thereby exacerbating the progression of secondary brain injury following acute brain injury diseases (5). The extracellular domain of LOX-1 is subjected to enzymatic hydrolysis, resulting in the release of sLOX-1 into the circulatory system. Monitoring the levels of sLOX-1 provides a means to gauge LOX-1 expression (6, 7). Experimental models of brain injury, including transient middle cerebral artery occlusion (tMCAO) and hypoxic-ischemic encephalopathy (HIE), have shown elevated LOX-1 expression to be associated with poor outcomes. Furthermore, serum sLOX-1 levels have been linked to injury severity and prognosis in both animal and neonatal studies, highlighting its potential as a biomarker for acute brain injuries (8–10). Thus, serum sLOX-1 may be a potential biomarker of acute brain injury. The study aims to evaluate the prognostic value of serum sLOX-1 levels in patients with sICH, focusing on their relationship with disease severity and functional outcomes.

## Materials and methods

### Study population

In this prospective observational cohort study, we consecutively enrolled patients with a primary diagnosis of sICH. Those patients were admitted to Neurosurgery Department at Lishui People’s Hospital within 24 h of the onset of stroke from March 2020 to January 2023. We required that sICH was confirmed through computed tomography (CT) plain scan, and all patients were aged older than 18 years, and undergo non-surgical treatments. The reasons for not performing the procedure include a small hematoma or the patient legal representatives’ refusal to perform the procedure.

Exclusion criteria were as follows: (a) recent surgical procedures or active infections within recent a month; (b) histories of neurological disorders, including ischemic stroke, hemorrhagic stroke, severe traumatic brain injury, Parkinson’s disease and Alzheimer’s disease; (c) secondary cerebral hemorrhages, such as ruptured intracranial aneurysms, intracranial tumors, arteriovenous malformations and ischemic strokes with hemorrhagic transformation; and (d) other systemic diseases, such as autoimmune diseases, uremia, cirrhosis, cancer and other severe chronic conditions. Additionally, a control group consisting of individuals without other diseases was recruited through health screening programs.

The study adhered to the ethical guidelines outlined by the World Medical Association (Declaration of Helsinki) and followed the ethical standards of our institution. Its protocol was approved by the Ethics Committee at the Lishui People’s Hospital (Opinion No. Medical Ethics Review No. 2020-001, 2020-002). Approval was obtained from the Ethics Committee of our institution, and written informed consent was acquired from the subjects or their relatives. Because patients with sICH were at state of consciousness disturbances or fluctuations, their legal representatives were informed of study details and authorized to sign written informed consent forms. And controls themselves provided written informed consent for willingness to participate in this study.

### Assessments and immune analysis

In this study, we collected a series of data, including basic information (such as age, gender, medical history, smoking and alcohol consumption), vital signs, time of admission, time of blood collection and biochemical data. Head CT scan was conducted and hematoma volume was calculated using the ABC/2 method (11). Disease severity was evaluated using the GCS scores and hematoma volumes. Functional outcomes at post-stroke 3 months were assessed using Glasgow Outcome Scale (GOS) scores. The GOS scores of 1–3 indicated a poor prognosis (12). Peripheral blood samples were obtained from sICH patients upon admission and centrifuged at 3,000 g for 15 min. The obtained serum specimens were stored at −80°C for subsequent testing. Serum sLOX-1 levels were determined using a commercially available enzyme-linked immunosorbent assay (Article No. YA-10765, Yilairui Biotechnology, Beijing). Its detection range is 1.0 pmol/mL–48.0 pmol/mL, the sensitivity is 1.0 pg/mL, and intra-assay coefficients of variation were <15% and inter-assay coefficients of variation were <15%. Notably, all tests were conducted by the same technician, who was blinded to the clinical data.

### Statistical analysis

All statistical analyses were performed using SPSS 25.0 (SPSS Inc., Chicago, Illinois, United States). Graphs were generated using GraphPad Prism 9.0 (GraphPad Software Inc., La Jolla, CA, United States) and MedCalc 9.6.4.0 (MedCalc Software, Mariakerke, Belgium). Continuous variables are presented as means (standard deviations) or medians (upper and lower quartiles), while categorical variables are expressed as counts (percentages). For the comparison of categorical variables, either the *χ*^2^ test or Fisher exact test was employed, whereas the Mann–Whitney *U* test or *t*-test was utilized for the comparison of continuous variables. The Kruskal–Wallis test was applied to compare serum sLOX-1 levels among different groups. Spearman correlation analysis or point-biserial correlation analysis were used for bivariate correlation test. Univariate and multifactorial correlation analyses were conducted using linear regression model, respectively. The binary logistic regression analysis was employed to identify independent predictors of a poor prognosis. To assess the predictive value of various serum sLOX-1 levels on the poor prognosis of sICH patients, receiver operating characteristic (ROC) curves were constructed, and the corresponding area under the curve (AUC) was estimated. The minimum sample size was estimated by G*Power 3.1.9.4 (Heinrich-Heine-Universität Düsseldorf, Universitätsstraße 1, Düsseldorf, Germany). For comparing differences of related variables between the two groups, the minimum sample size was estimated to be 47 cases and the actual power is 0.953. Thus, a total of 118 sICH patients and 118 healthy controls were finally included in this study, meeting the required sample size. Two-sided *p* < 0.05 indicates significant differences.

## Result

### Clinical features of sICH patients and controls

**Figure 1:**
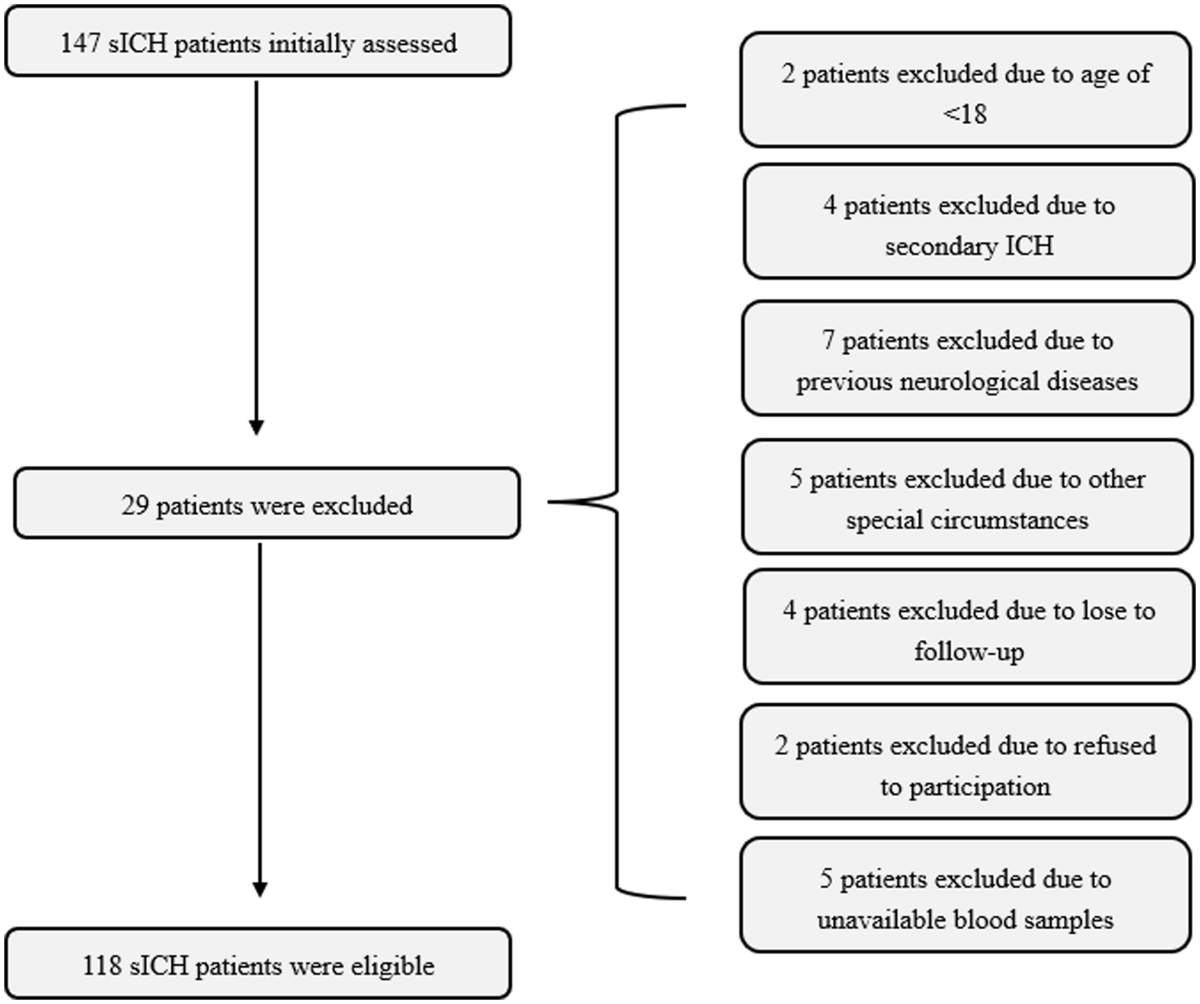
Flowing-chart for selecting eligible patients with sICH. Initially, a total of 147 patients were assessed and ultimately, 118 patients were analyzed after excluding 29 patients in compliance with the exclusion criteria. sICH, spontaneous intracerebral hemorrhage.

In our study, a total of 147 patients with sICH were initially enrolled in the study. After excluding 29 patients for reasons detailed in Figure 1, the final cohort consisted of 118 patients, who comprised 68 males and 50 females, with age ranging from 33 to 89 years (mean age, 64.5 years; standard deviation, 12.5 years). Additionally, 118 healthy volunteers were recruited as controls, including 71 males and 47 females, with ages ranging from 32 to 83 years (mean age, 61.0 years; standard deviation, 11.4 years). The mean age and gender ratio in the control group were similar to those in the patient group (both *p* > 0.05).

Among this cohort of patients, 25 (21.5%) were smokers, 30 (25.4%) were alcohol drinkers, 79 (66.9%) suffered from hypertension, 23 (19.5%) had diabetes mellitus, and 34 (28.8%) were inflicted with hyperlipidemia. Duration of hospitalization ranged from 1.0 to 23.0 h after stroke (median 6.0 h; 25th–75th, 4.0–11.3 h), time to blood collection ranged from 1.2 to 25.1 h (median, 6.7 h; 25th–75th, 4.8–11.4 h), and systolic blood pressure ranged from 96 to 221 mmHg (mean, 158.0 mmHg; standard deviation, 20.2 mmHg) and diastolic blood pressure ranged from 59–120 mmHg (mean, 88.6 mmHg; standard deviation, 14.4 mmHg). Blood leukocyte counts ranged from 4.5–16.0 × 10^9^/L (median, 8.7 × 10^9^/L; 25th–75th, 6.7–10.8 × 10^9^/L), and blood glucose levels ranged from 4.7–19.2 mmol/L (median, 8.0 mmol/L; 25th–75th, 6.4–10.3 mmol/L), serum potassium levels ranged from 2.76–5.41 mmol/L (mean, 3.74 mmol/L, standard deviation, 0.48 mmol/L). GCS scores ranged from 4–15 (median, 12; 25th–75th, 7–13), and hematoma volume ranged from 2.9–58.8 mL (median, 18.1 mL; 25th–75th, 10.8–30.6 mL). Meanwhile, a total of 17 (14.4%) sICH patients with intraventricular hemorrhage were included in the study.

### Serum sLOX-1 levels and severity of sICH

**Figure 2:**
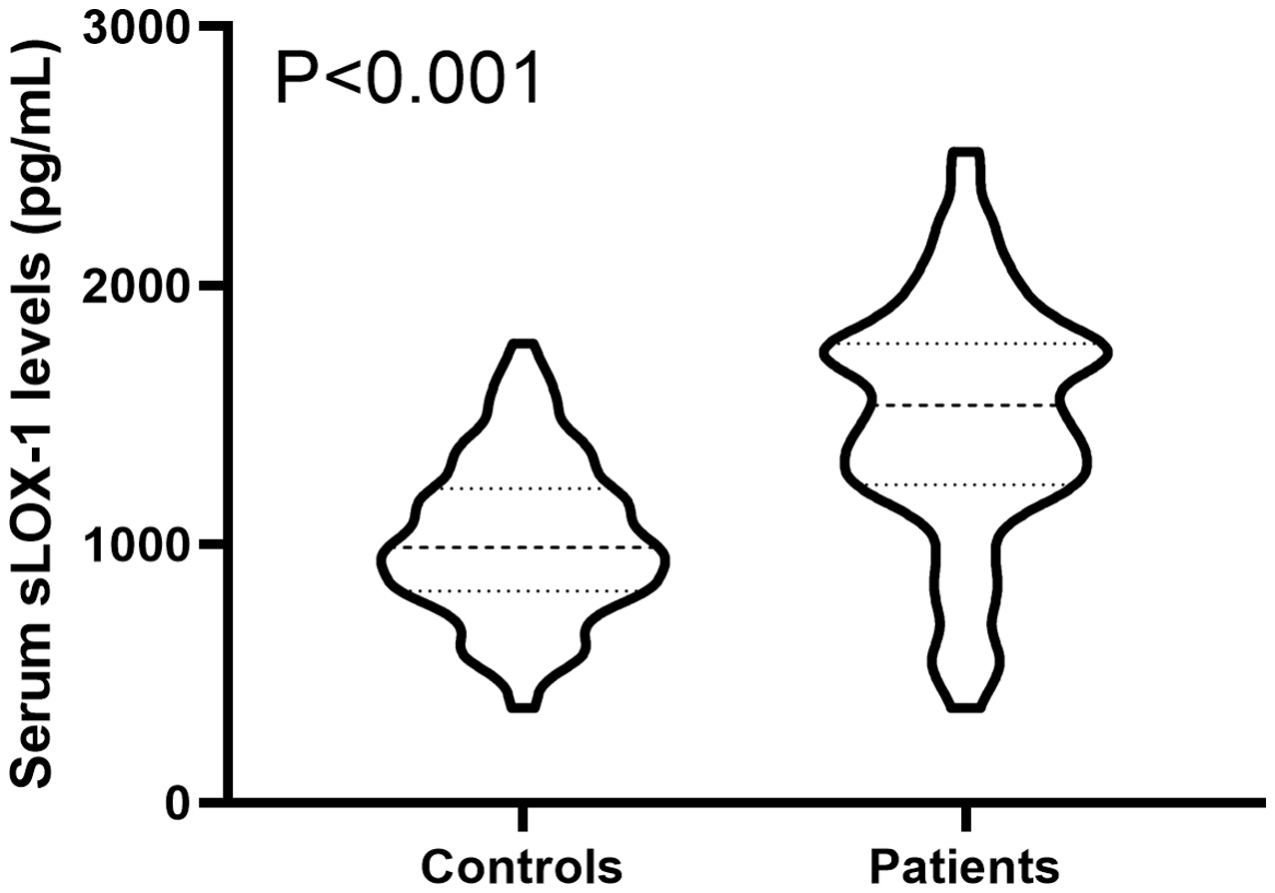
Boxplot illustrating admission serum sLOX-1 levels between patients with sICH and controls. Data were reported as median (25th–75th) and two groups were compared using the Mann–Whitney *U*-test. Serum soluble lectin-like oxidized low-density lipoprotein receptor-1 levels at admission were statistically significant higher in patients with sICH than in controls (*p* < 0.001). sLOX-1, soluble lectin-like oxidized low-density lipoprotein receptor-1; sICH, spontaneous intracerebral hemorrhage.

**Figure 3:**
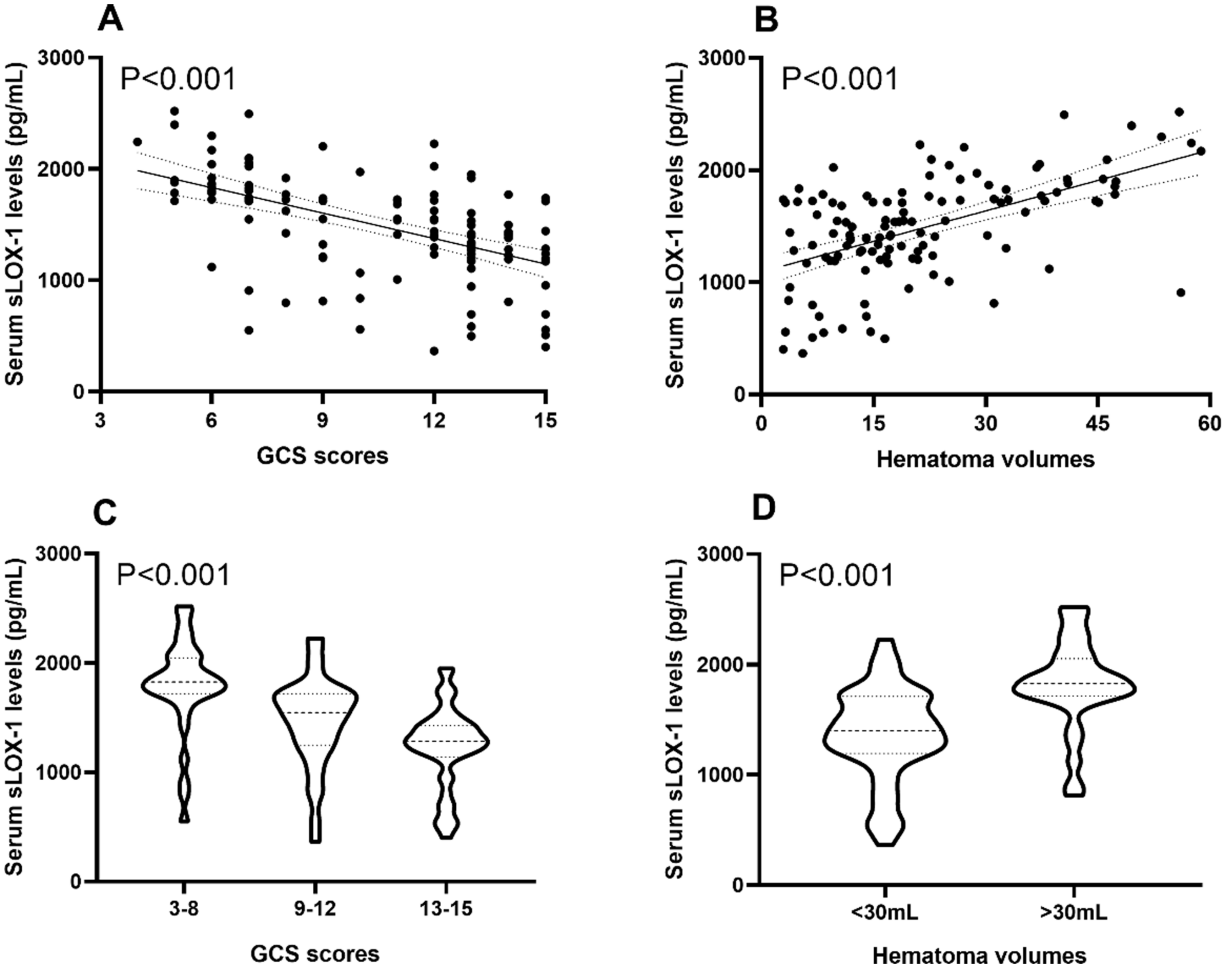
Boxplots and correlograms describing relationships between admission serum sLOX-1 levels and disease severity of sICH. **(A)** Correlograms illustrating the relationship between admission serum sLOX-1 levels and baseline GCS scores after sICH using the Spearman correlation coefficient. **(B)** Correlograms illustrating the relationship between admission serum sLOX-1 levels and baseline hematoma volumes after sICH using the Spearman correlation coefficient. **(C)** Boxplots illustrating admission serum sLOX-1 levels among subgroups based on baseline GCS scores after sICH. **(D)** Boxplots illustrating admission serum sLOX-1 levels among subgroups based on baseline hematoma volumes after sICH. sLOX-1, soluble lectin-like oxidized low-density lipoprotein receptor-1; GCS, Glasgow Coma Scale; sICH, spontaneous intracerebral hemorrhage.

In Figure 2, serum sLOX-1 levels were statistically significant higher in patients than in controls. In Table 1, Spearman’s correlation analysis showed that serum sLOX-1 levels were significantly correlated with some continuous variables, such as age, GCS score, hematoma volumes, blood glucose levels and blood leukocyte count. And as show in Table 2, point-biserial correlation analysis shows that serum sLOX-1 levels were significantly correlated with intraventricular hemorrhage. Interestingly, in Table 3, one-way linear regression analysis showed similar results. Then, we included those variables in a multifactorial linear regression model and found that GCS scores and hematoma volumes were independently correlated with serum sLOX-1 levels. Figure 3 shows the close relationship between serum sLOX-1 levels and GCS scores plus hematoma volumes, when considering them as either the continuous or categorical variables.

**Table 1:** Correlation between serum soluble lectin-like oxidized low-density lipoprotein receptor-1 levels and other continuous variables using Spearman’s correlation coefficient in acute intracerebral hemorrhage.

Variables	*ρ*	*p*-value
Age (years)	0.291	0.001
Admission time (h)	0.016	0.864
Blood-collection time (h)	0.002	0.985
Systolic arterial pressure (mmHg)	−0.017	0.857
Diastolic arterial pressure (mmHg)	−0.042	0.654
GCS scores	−0.577	<0.001
Hematoma volumes (mL)	0.540	<0.001
Blood leucocyte count (×10^9^/L)	0.192	0.038
Blood potassium levels (mmol/L)	−0.144	0.120
Blood glucose levels (mmol/L)	0.272	0.003

Correlations were done using Spearman correlation coefficient in intracerebral hemorrhage. GCS, Glasgow Coma Scale.

**Table 2:** Correlation between serum soluble lectin-like oxidized low-density lipoprotein receptor-1 levels and other binary categorical variables using point-biserial correlation analysis in acute intracerebral hemorrhage.

Variables	*r*	*p*-value
Gender (male/female)	0.003	0.978
Hypertension	−0.019	0.839
Diabetes mellitus	0.144	0.120
Hyperlipidemia	−0.001	0.994
Current smoking	0.117	0.205
Alcohol consumption	0.059	0.525
Intraventricular hemorrhage	0.195	0.034

Correlations were done using point-biserial correlation analysis in intracerebral hemorrhage. GCS, Glasgow Coma Scale.

**Table 3:** Multivariate linear regression analysis between elevated serum soluble lectin-like oxidized low-density lipoprotein receptor-1 levels and other variables.

Variables	Univariate analysis		Multivariate analysis	
	*t*	*p*-value	*t*	*p*-value
Age (years)	2.984	0.003	1.197	0.234
Gender (male/female)	0.028	0.978	—	—
Hypertension	−0.204	0.839	—	—
Diabetes mellitus	1.582	0.116	—	—
Hyperlipidemia	−0.007	0.994	—	—
Current smoking	1.273	0.205	—	—
Alcohol consumption	0.637	0.525	—	—
Admission time (h)	0.923	0.358	—	—
Blood-collection time (h)	0.763	0.447	—	—
Systolic arterial pressure (mmHg)	−0.352	0.726	—	—
Diastolic arterial pressure (mmHg)	−0.552	0.582	—	—
Intraventricular hemorrhage	2.144	0.034	−1.221	0.225
GCS scores	−6.732	<0.001	−2.484	0.014
Hematoma volumes (mL)	7.136	<0.001	2.709	0.008
Blood leucocyte count (×10^9^/L)	2.709	0.008	1.728	0.087
Blood glucose levels (mmol/L)	3.517	0.001	0.756	0.451
Blood potassium levels (mmol/L)	−1.599	0.113	—	—

Correlations was analyzed using multivariate linear regression model. GCS, Glasgow Coma Scale.

### Serum sLOX-1 levels and poor 90-day prognosis after sICH

**Figure 4:**
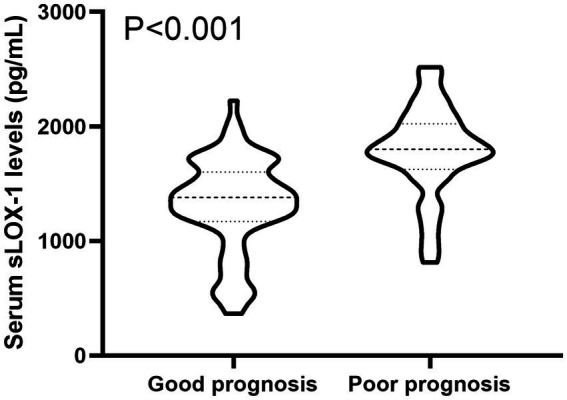
Boxplot illustrating admission serum sLOX-1 levels between patients suffering with good prognosis and those with poor prognosis. Data were reported as median (25th–75th) and two groups were compared using the Mann–Whitney *U*-test. Serum sLOX-1 levels at admission were statistically significant higher in patients with sICH than in controls (*p* < 0.001). sLOX-1, soluble lectin-like oxidized low-density lipoprotein receptor-1; sICH, spontaneous intracerebral hemorrhage.

**Figure 5:**
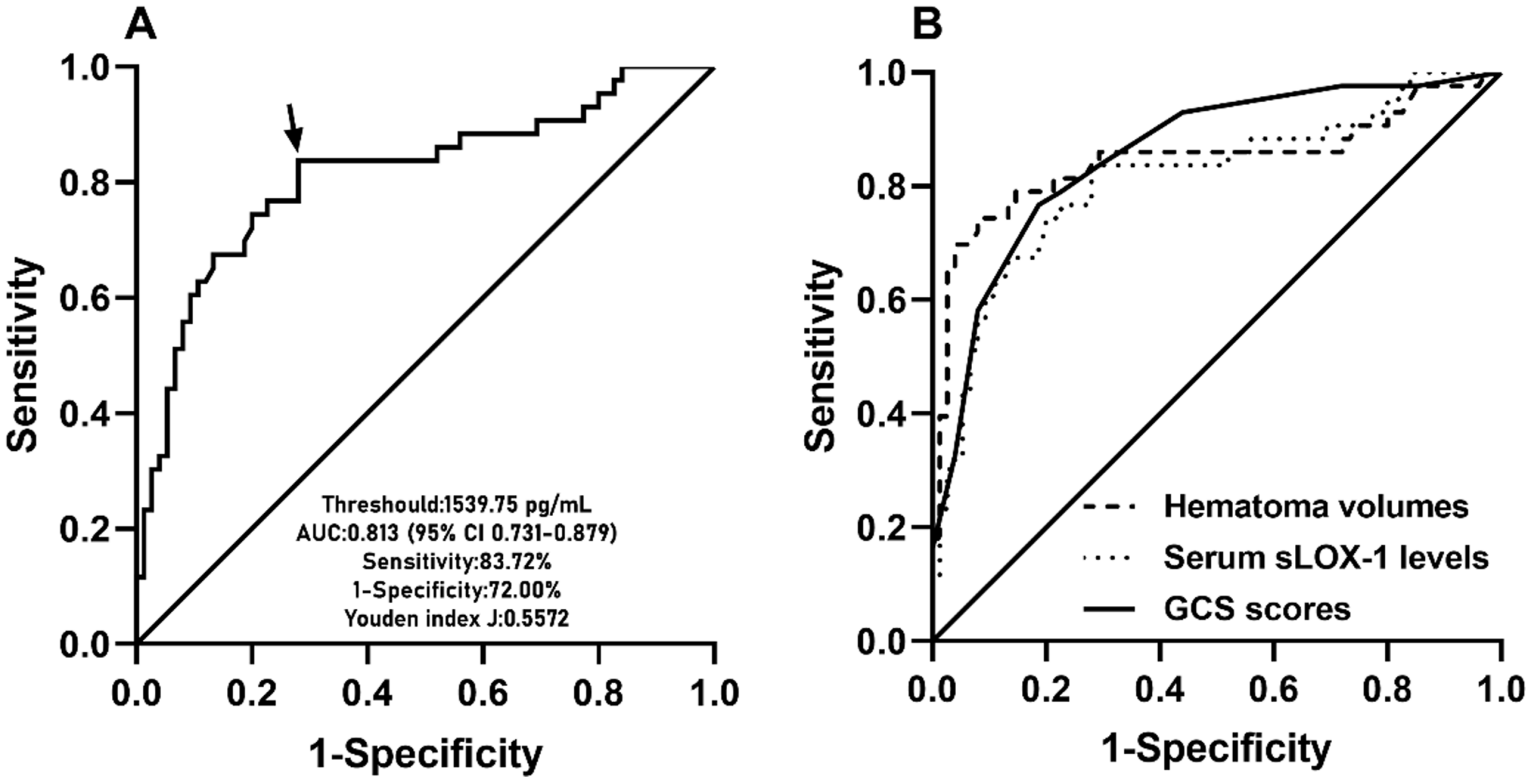
ROC curve of admission serum sLOX-1 levels with respect to 90-day poor prognosis predictive ability among patients with sICH. **(A)** ROC curve for admission serum sLOX-1 levels used to predict 90-day poor prognosis after sICH. Admission serum sLOX-1 levels significantly predicted 90-day poor prognosis after sICH (*p* < 0.001). Its optimal level was identified, which predicted 90-day poor prognosis with the maximum Youden index. **(B)** ROC curve for admission serum sLOX-1 levels, baseline GCS scores and baseline hematoma volumes used to predict 90-day poor prognosis after sICH. The post-stroke 90-day poor prognosis predictive ability of admission serum sLOX-1 levels was similar to those of baseline GCS scores and baseline hematoma volumes (both *p* > 0.05). sLOX-1, soluble lectin-like oxidized low-density lipoprotein receptor-1; GCS, Glasgow Coma Scale; AUC, area under curve; ROC, receiver operating characteristic; sICH, spontaneous intracerebral hemorrhage.

A total of 43 patients (36.4%) had a poor prognosis (GOS scores of 1–3) at 3 months after sICH. In Table 4, GOS scores were significantly correlated with age, GCS scores, hematoma volumes, blood glucose levels, blood leukocyte count and serum sLOX-1 levels. And as shown in Table 5, point-biserial correlation analysis shows that GOS scores were significantly correlated with intraventricular hemorrhage. Subsequently, the multifactor linear regression (Table 6) showed that GCS scores, hematoma volumes and serum sLOX-1 levels were independently correlated with GOS scores at 3 months after stroke. As shown in Figure 4, serum sLOX-1 levels were statistically significant higher in patients with poor prognosis than in patients with good prognosis. In Table 7, age, GCS scores, hematoma volumes, intraventricular hemorrhage, blood glucose levels, blood leukocyte count, and serum sLOX-1 levels were statistically significant different between the two groups. Subsequent univariate Logistic regression model showed similar results. In Table 8, when incorporating the above variables into the multivariate Logistic regression model, it was found that GCS scores, hematoma volumes, and serum sLOX-1 levels were independently associated with poor prognosis at 3 months after stroke. Figure 5A showed that serum sLOX-1 levels significantly predicted poor prognosis at 3 months with AUC of 0.813 (95% CI, 0.731–0.879). In Figure 5B, the predictive ability of serum sLOX-1 levels was similar to GCS scores (AUC = 0.857; 95% CI, 0.781–0.915; *p* = 0.355) and hematoma volumes (AUC = 0.846; 95% CI, 0.768–0.906; *p* = 0.580).

**Table 4:** Correlation between Glasgow Outcome Scale scores and other continuous variables using Spearman’s correlation coefficient in spontaneous intracerebral hemorrhage.

Variables	*ρ*	*p*-value
Age (years)	−0.318	<0.001
Admission time (h)	0.175	0.059
Blood-collection time (h)	0.171	0.064
Systolic arterial pressure (mmHg)	−0.049	0.598
Diastolic arterial pressure (mmHg)	−0.016	0.859
GCS scores	0.629	<0.001
Hematoma volumes (mL)	−0.648	<0.001
Blood leucocyte count (×10^9^/L)	−0.241	0.008
Blood glucose levels (mmol/L)	−0.305	0.001
Blood potassium levels (mmol/L)	0.076	0.411
Serum sLOX-1 levels (pg/mL)	−0.539	<0.001

Correlations were done using Spearman correlation coefficient in intracerebral hemorrhage. GCS, Glasgow Coma Scale; sLOX-1, soluble lectin-like oxidized low-density lipoprotein receptor-1. The asterisk indicates statistical significance (^*^p < 0.05).

**Table 5:** Correlation between Glasgow Outcome Scale scores and other binary categorical variables using point-biserial correlation analysis in acute intracerebral hemorrhage.

Variables	*r*	*p*-value
Gender (male/female)	−0.003	0.973
Hypertension	−0.143	0.122
Diabetes mellitus	−0.131	0.159
Hyperlipidemia	−0.099	0.287
Current smoking	−0.169	0.067
Alcohol consumption	−0.023	0.804
Intraventricular hemorrhage	−0.431	<0.001

Correlations were done using point-biserial correlation analysis in intracerebral hemorrhage. GCS, Glasgow Coma Scale.

**Table 6:** Multivariate linear regression analysis between Glasgow Outcome Scale scores and other variables.

Variables	Univariate analysis		Multivariate analysis	
	*t*	*p*-value	*t*	*p*-value
Age (years)	−2.660	0.009	−0.853	0.395
Gender (male/female)	−0.034	0.973	—	—
Hypertension	−1.560	0.122	—	—
Diabetes mellitus	−1.418	0.159	—	—
Hyperlipidemia	−0.513	0.609	—	—
Current smoking	−1.848	0.067	—	—
Alcohol consumption	−0.248	0.804	—	—
Admission time (h)	1.295	0.198	—	—
Blood-collection time (h)	1.269	0.207	—	—
Systolic arterial pressure (mmHg)	−0.730	0.467	—	—
Diastolic arterial pressure (mmHg)	0.266	0.791	—	—
Intraventricular hemorrhage	−4.723	<0.001	−1.645	0.103
GCS scores	9.492	<0.001	2.538	0.012
Blood C-creation protein levels (mg/mL)	−2.765	0.007	−0.110	0.912
Hematoma volumes (mL)	−10.199	<0.001	−2.974	0.004
Blood leucocyte count (×10^9^/L)	−2.522	0.013	−1.007	0.316
Blood glucose levels (mmol/L)	−5.194	<0.001	−1.219	0.225
Blood potassium levels (mmol/L)	−0.784	0.434	—	—
Serum sLOX-1 levels (pg/mL)	−7.467	<0.001	−2.412	0.017

Correlations was analyzed using multivariate linear regression model. GCS, Glasgow Coma Scale; sLOX-1, soluble lectin-like oxidized low-density lipoprotein receptor-1. The asterisk indicates statistical significance (^*^p < 0.05).

**Table 7:** Factors associated with 3-month poor prognosis in spontaneous intracerebral hemorrhage.

Variables	Poor prognosis	Good prognosis	*t*/*χ*^2^/*Z*	*p*-value
Age (years)	70.6 ± 13.3	64.0 ± 11.9	−2.740	0.007
Gender (male/female)	25/18	43/32	0.007	0.932
Hypertension	31 (72.1%)	48 (64.0%)	0.809	0.368
Diabetes mellitus	10 (23.3%)	13 (17.3%)	0.611	0.434
Hyperlipidemia	17 (39.5%)	17 (22.7%)	3.791	0.052
Current smoking	12 (27.9%)	13 (17.3%)	1.830	0.176
Alcohol consumption	12 (27.9%)	18 (24.0%)	0.220	0.639
Admission time (h)	5.0 (3.0–11.0)	6.0 (5.0–12.0)	−1.264	0.206
Blood-collection time (h)	6.1 (4.1–11.3)	7.1 (5.5–11.7)	−1.186	0.236
Systolic arterial pressure (mmHg)	161.3 ± 21.9	156.3 ± 19.3	−1.280	0.203
Diastolic arterial pressure (mmHg)	89.6 ± 15.8	88.4 ± 14.2	−0.452	0.652
Intraventricular hemorrhage	14 (32.6%)	3 (4.0%)	18.076	<0.001
GCS score	7 (6–9)	13 (11–14)	−6.486	<0.001
Hematoma volume (mL)	33.0 (22.7–45.1)	14.6 (9.5–19.0)	−6.241	<0.001
Blood leucocyte count (×10^9^/L)	9.8 (7.3–11.5)	7.9 (6.5–9.7)	−2.606	0.009
Blood glucose levels (mmol/L)	9.7 (6.7–14.0)	7.6 (6.2–9.5)	−2.835	0.005
Blood potassium levels (mmol/L)	3.75 ± 0.54	3.67 ± 0.37	−0.925	0.357
Serum sLOX-1 levels (pg/mL)	1800.15 (1624.01–2022.80)	1379.99 (1170.70–1602.56)	−5.651	<0.001

Qualitative variables were presented as counts (percentages) and were compared for intergroup difference using chi-square test or Fisher exact test as appropriate. Quantitative variables were summarized as medians (lower-upper quartiles) if non-normally distributed and were presented as means ± standard deviations if normally distributed. Intergroup comparisons were done using unpaired Student *t*-test or Mann–Whitney *U* test where appropriate. GCS, Glasgow Coma Scale; sLOX-1, soluble lectin-like oxidized low-density lipoprotein receptor-1. Glasgow Outcome Scale scores of 1–3 was designated as poor prognosis.

**Table 8:** Univariate and multivariate logistic regression analysis of predictors for 3-month poor outcome after intracerebral hemorrhage.

Variables	Univariate analysis		Multivariate analysis	
	Odds ratio (95% CI)	*p*-value	Odds ratio (95% CI)	*p*-value
Age (years)	1.047 (1.012–1.083)	0.009	1.008 (0.962–1.057)	0.738
Gender (male/female)	0.968 (0.453–2.067)	0.932	—	—
Hypertension	1.453 (0.642–3.287)	0.370	—	—
Diabetes mellitus	1.445 (0.572–3.649)	0.436	—	—
Hyperlipidemia	2.231 (0.986–5.045)	0.054	—	—
Current smoking	1.846 (0.754–4.519)	0.180	—	—
Alcohol consumption	1.226 (0.523–2.872)	0.639	—	—
Admission time (h)	0.972 (0.905–1.043)	0.426	—	—
Blood-collection time (h)	0.974 (0.908–1.044)	0.452	—	—
Systolic arterial pressure (mmHg)	1.012 (0.993–1.032)	0.212	—	—
Diastolic arterial pressure (mmHg)	1.005 (0.979–1.031)	0.723	—	—
Intraventricular hemorrhage	11.586 (3.097–43.344)	<0.001	5.554 (0.839–36.761)	0.075
GCS score	0.603 (0.507–0.717)	<0.001	0.784 (0.622–0.988)	0.039
Hematoma volume (mL)	1.141 (1.088–1.198)	<0.001	1.060 (1.002–1.121)	0.042
Blood leucocyte count (×10^9^/L)	1.215 (1.046–1.412)	0.011	1.224 (0.974–1.538)	0.083
Blood glucose levels (mmol/L)	1.240 (1.097–1.402)	<0.001	1.036 (0.859–1.250)	0.712
Blood potassium levels (mmol/L)	1.053 (0.481–2.307)	0.355	—	—
Serum sLOX-1 levels (pg/mL)	1.003 (1.002–1.005)	<0.001	1.002 (1.000–1.004)	0.037

Results were presented as odds ratio (95% confidence interval) using the univariate and multivariate logistic regression analysis. 95% CI, confidence interval; GCS, Glasgow Coma Scale; sLOX-1, soluble lectin-like oxidized low-density lipoprotein receptor-1. Glasgow Outcome Scale scores of 1–3 was designated as poor prognosis.

**Figure 6:**
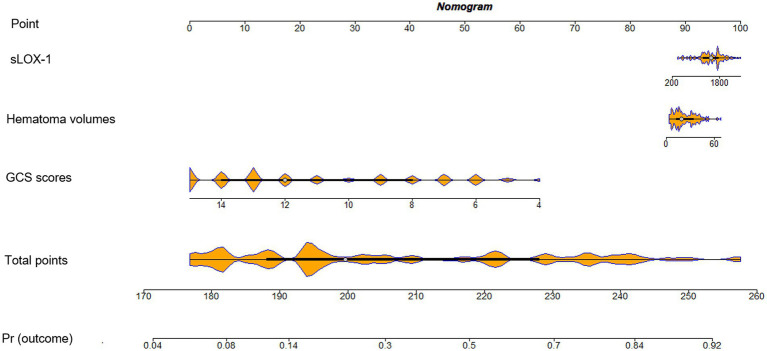
Nomogram assessing risk of poor prognosis after sICH. sLOX-1, soluble lectin-like oxidized low-density lipoprotein receptor-1; sICH, spontaneous intracerebral hemorrhage.

**Figure 7:**
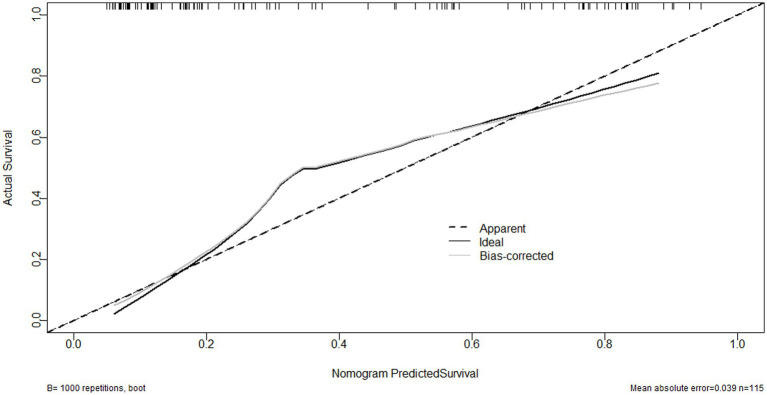
Calibration curve showing stability of the model for predicting poor prognosis following sICH. sICH, spontaneous intracerebral hemorrhage.

**Figure 8:**
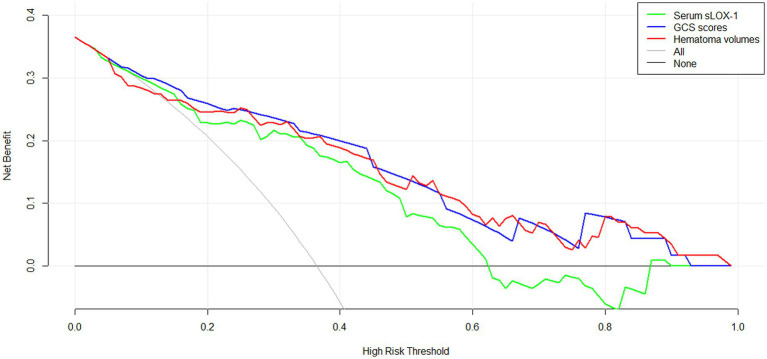
Decision curve displaying clinical fit of the model for predicting poor prognosis following sICH. sLOX-1, soluble lectin-like oxidized low-density lipoprotein receptor-1, GCS, Glasgow Coma Scale; sICH, spontaneous intracerebral hemorrhage.

As shown in Figure 6, serum sLOX-1 levels, GCS scores, and hematoma volumes were forced into the nomogram model to predict the associated risk. The points corresponding to the above three variables were summed to calculate the total points, and different points corresponded to different survival probabilities, which were 0.1 = 18.1, 0.3 = 49.1, 0.5 = 68.8, 0.7 = 88.1, and 0.9 = 119.1, respectively. In addition, Figure 7 showed that the model was stable in the context of the calibration curve assessment. In Figure 8, decision curves were established, suggesting that the model has a high clinical value.

## Discussion

sICH is a common type of stroke for its rapid onset, swift progression, and often unfavorable prognosis. The timely diagnosis and accurate prognosis prediction of ICH are crucial for effective treatments. Numerous studies have demonstrated elevated circulating levels of sLOX-1 following acute ischemic stroke and aneurysmal subarachnoid hemorrhage (13, 14). In clinical practice, GCS scores and hematoma volumes serve as pivotal indicators for evaluating disease severity following sICH and predicting neurological sequelae. Over recent decades, scholars have directed their attention towards elucidating the prognostic predictive capacity of peripheral blood biomarkers in the context of sICH. In our current investigation, we observed a significant elevation in serum sLOX-1 levels during the early stages of sICH. Notably, our findings revealed additional intriguing insights: (1) there was a significant negative correlation between serum sLOX-1 levels and patients’ GCS scores, alongside a significant positive correlation with hematoma volumes; (2) serum sLOX-1 was identified as an independent predictor of poor prognosis at 3 months post-stroke; (3) there was a high predictive ability of serum sLOX-1 levels for poor prognosis in sICH; and (4) the predictive ability of serum sLOX-1 levels for poor prognosis were similar to those of GCS scores and hematoma volumes. Consequently, serum sLOX-1 may emerge as a potential biomarker for evaluating disease severity and predicting an unfavorable prognosis in patients with sICH.

Several studies have underscored the significance of secondary brain injury in determining the prognosis of patients with acute brain injury diseases. The mechanisms driving secondary brain injury encompass inflammatory responses, oxidative stress, cytotoxic reactions, blood–brain barrier disruption, and neuronal apoptosis (15). This intricate pathophysiological process not only exacerbates brain tissue damage but also elevates the likelihood of an unfavorable prognosis.

LOX-1 is a member of the C-type hemagglutinin family, which is initially identified on aortic endothelial cells (16). Accumulating studies have demonstrated that LOX-1 plays a pivotal role in mediating endothelial cell endocytosis, facilitating the degradation of Ox-LDL and instigating various vascular inflammatory responses. This sequence of events contributes to endothelial cell dysfunction, thereby not only contributing to the development of diverse diseases, including acute stroke, but also exacerbating the process of secondary brain injury following acute brain injury diseases (5). The extracellular portion of LOX-1 undergoes enzymatic hydrolysis, giving rise to a soluble form known as sLOX-1 in the circulatory system. Consequently, the levels of sLOX-1 in the circulatory system may serve as a reflective measure of LOX-1 expression (6, 7).

The mechanisms, through which LOX-1 is implicated in acute brain injury diseases, remain not fully elucidated. In a rat model of transient focal cerebral ischemia, a study analyzing gene expression changes identified seven differentially expressed genes, with LOX-1 showing a significant elevation at the site of ischemic injury (17). Furthermore, in a rat model of ICH, the knockdown of the LOX-1 gene exhibited a protective effect against brain injury, indicating LOX-1’s involvement in the pathophysiological process of blood-brain barrier disruption post-cerebral hemorrhage (18). Notably, a study conducted in mice revealed elevated LOX-1 expression in the brain tissue of the transient middle cerebral artery occlusion mouse model and its detrimental effects on acute brain injury (8). In a rat model of HIE, scholars found that anti-LOX-1 neutralizing antibody treatment reduced infarct size, brain edema, and apoptotic cell death (9). Subsequent research involving 386 patients with cerebral infarction revealed significantly higher CC + GC genotype, GC genotype, and C allele frequency of the LOX-1 expression-related gene G501C in patients with cerebral infarction compared to controls (19). These findings suggest that LOX-1 could be a potential therapeutic target of acute brain injury.

Several clinical studies have explored the correlation between circulating sLOX-1 levels and the severity as well as the prognosis of acute brain injury diseases. In one study involving 378 patients with ischemic stroke and 377 patients with stroke (comprising 250 patients with ischemic stroke and 127 patients with hemorrhagic stroke), plasma sLOX-1 levels were significantly elevated in the patient group compared to the control group (14, 20). Additionally, a study focusing on 148 patients with acute ischemic stroke of the LAA subtype revealed significantly increased serum sLOX-1 levels in the patient group. Importantly, these levels exhibited a robust correlation with disease severity, and patients with a good prognosis exhibited significantly lower serum sLOX-1 levels compared to those with a poor prognosis (21). In a study involving 94 patients with aneurysmal subarachnoid hemorrhage (aSAH), serum sLOX-1 levels were significantly higher in patients with aSAH and exhibited a significant correlation with the patients’ WFNS classification, Hunt–Hess score, and modified Fisher score. Elevated sLOX-1 levels served as a significant predictor of poor prognosis in those patients (22). This trend was further supported by another study with 125 patients with aSAH, where serum sLOX-1 levels displayed a significant positive correlation with the severity of bleeding. Moreover, the levels were strongly associated with the development of delayed cerebral ischemia in patients (13). These findings collectively underscore the potential of circulating sLOX-1 levels as a valuable biomarker for assessing the severity and predicting the prognosis of acute brain injury diseases.

In our study, we enrolled a total of 118 sICH patients. Notably, we observed statistically significant higher serum sLOX-1 levels in patients after sICH, compared to healthy controls. Spearman’s rank correlation test revealed a significant negative correlation between patients’ serum sLOX-1 levels and GCS scores, alongside a significant positive correlation with hematoma volumes. Subsequent multiple linear regression analysis confirmed the independent correlation of serum sLOX-1 levels with these parameters, supporting the potential of sLOX-1 as an indicator of bleeding severity in sICH patients. Although age, intraventricular hemorrhage, blood glucose levels, and blood leukocyte count are significantly correlated with serum sLOX-1 levels on univariate analysis, but not on multivariate analysis, meaning that the above four factors could not obviously affect serum sLOX-1 levels after ICH. In addition, our multiple Logistic regression analysis demonstrated a significant association between patients’ serum sLOX-1 levels and poor prognosis at 3 months post-stroke, establishing sLOX-1 as an independent predictor of 3-month poor prognosis. Notably, serum sLOX-1 levels exhibited significant prognostic accuracy in identifying 3-month prognostic prognosis after stroke, as reflected in the ROC curve. Intriguingly, when we compared the discriminatory ability of serum sLOX-1 levels with that of GCS scores and hematoma volumes, we found a similar predictive ability for long-term poor prognosis after stroke. In conclusion, our findings suggest that serum sLOX-1 has the potential to serve as a biomarker for assessing disease severity and predicting long-term prognosis in patients with sICH.

## Conclusion

In this prospective observational cohort study, we investigate the correlations between serum sLOX-1 levels, disease severity, and the 3-month adverse prognosis in patients with sICH. Our analysis reveal a strong association of elevated serum sLOX-1 levels, GCS scores, and hematoma volumes with patients’ poor prognosis. Importantly, these factors emerge as independent predictors of a 3-month poor prognosis, demonstrating comparable prognostic ability. In summary, our findings suggest that serum sLOX-1 levels may hold promise as a potential biomarker for assessing disease severity and predicting poor prognosis in patients with sICH.

### Limitations

The present study has several limitations. First, this study demonstrated the predictive role of serum sLOX-1 levels for poor prognosis after sICH, but the sample size was moderate. Therefore, further cohort studies with larger sample sizes are needed to demonstrate the current findings. Second, multiple comparisons, the best of our knowledge, are scarcely used in multivariate analysis of prospective cohort studies, maybe because prospective cohort studies are often characterized by a small sample size. However, the multiple comparisons merit utilization in future larger-cohort studies for validating the conclusions in the current study. Third, ELISA detected serum sLOX-1 levels in the present study, and more sensitive LC-MS techniques could be used to detect serum sLOX-1 levels in subsequent studies. Fourth, the present study detected serum sLOX-1 levels only in the early stage of the disease in patients with sICH; therefore, it may be clinically relevant to study its dynamic changes. Finally, GOS scores at 3 months after sICH could not be recorded for four patients lost to follow-up and therefore these patients were not included in the 3-month function prognosis analysis. Although only four cases were lost to follow-up, the exclusion of patients with missed visits may have an impact on the results of the experiment statistically.

## Data Availability

The original contributions presented in the study are included in the article/supplementary material, further inquiries can be directed to the corresponding author.
